# Supplementary material for: Dose Prediction and Pharmacokinetic Simulation of XZP-5610, a Small Molecule for NASH Therapy, Using Allometric Scaling and Physiologically Based Pharmacokinetic Models
Source: Pharmaceuticals (Basel). 2024 Mar 13;17(3):369. doi: 10.3390/ph17030369 (PMC10975475; doi:10.3390/ph17030369)
Supplement: Supplementary file 1 [file pharmaceuticals-17-00369-s001.zip › pharmaceuticals-2881873-supplementary.pdf]

1. Calculation of hepatic clearance of XZP-5610

$$\%Remaining = \frac{Peak\ area\ ratio\ of\ reference\ to\ internal\ standard\ at\ any\ point}{Peak\ area\ ratio\ of\ reference\ to\ internal\ standard\ at\ 0\ min} \times 100 \quad (1)$$

$$T_{1/2}=0.693/k \quad (2)$$

$$CL_{int(hep)} = \frac{k}{million\ cells/mL} \quad (3)$$

$$CL_{int(liver)} = CL_{int(hep)} \times cells\ per\ g\ liver \times Liver/Body\ weight \quad (4)$$

Where k is the elimination rate constant, which was calculated as the slope of time against the logarithm of remaining. $CL_{int(hep)}$ is the intrinsic clearance in hepatocytes, $T_{1/2}$ is the half-life in hepatocytes. $CL_{int(liver)}$ is the intrinsic clearance in liver. The parameters used for calculation were from references and listed as follow.

Species	Liver weight/body weight (g/kg Body Weight) [22-23]	Liver blood flow (Qh) (mL/min/kg) [22-23]	Hepatocytes content (of cells/g liver) [24]
Mice	88	90.0	135×10^6
Rat	40	55.2	117×10^6
Beagle	32	30.9	215×10^6
Cynomolgus	30	43.6	120×10^6
Human	20	20.7	139×10^6

2. Calculation of human $CL_{i.v}$ and V_{ss} of XZP-5610

The calculation methods of $CL_{i.v}$ were as follows:

Single-species scaling method:

$$CL_{i.v,h} = a \times CL_{i.v,a} \times \frac{BW_h}{BW_a} \quad (5)$$

Single species allometric scaling method:

$$CL_{i.v,h} = \frac{CL_{i.v,a}}{f_{u.a}} \times \frac{BW_h}{BW_a}^{0.75} \times f_{u,h} \quad (6)$$

Two species allometric scaling method:

$$CL_{i.v,h} = b \times BW_h^{0.628} \quad (7)$$

f_u corrected intercept method:

$$CL_{i.v,h} = 33.35 \times \frac{b}{Rf_{up}}^{0.77} \times mL/min \quad (8)$$

Hepatic blood flow method:

$$CL_{i.v,h} = CL_{i.v,a} \times \frac{Q_h}{Q_a} \quad (9)$$

Where $CL_{i.v,h}$ represents human intravenous clearance rate; $CL_{i.v,a}$ represents animal intravenous clearance rate; BW_h represents human body weight, which is 60 kg; BW_a represents animal body weight, for SD rat is 0.245 kg, and for beagle dog is 7.21 kg; a represents the allometric scaling factor, for SD rat is 0.152, and for beagle dog is 0.41; b represents the intercept, which is calculated based on the regression of $Ln_{(CL)}$ and $Ln_{(WT)}$ from different animal species experiments, in this study it was 13.40 as

calculated; $f_{u,a}$ represents animal plasma free fraction; $f_{u,h}$ represents human plasma free fraction; Rf_{up} represents the ratio of animal plasma free fraction to human plasma free fraction; Q_h is the hepatic blood flow for humans, which is 1450 mL/min; Q_a is the hepatic blood flow for animals, which is 13.5 mL/min for SD rat and 222.8 mL/min for beagle dog.

The calculation methods of V_{ss} were as follows:

Single-species scaling method:

$$V_{ss,h} = a \times V_{ss,a} \times \frac{BW_h}{BW_a} \quad (10)$$

Øie-Tozer method:

$$V_{ss,h} = V_p \times (1 + R_{E/I}) + f_{up} \times V_p \times \left(\frac{V_E}{V_p} - R_{E/I}\right) + \frac{V_R \times f_{up}}{f_{ut}}$$

where f_{up} is the fraction unbound in plasma, f_{ut} is the fraction unbound in tissues, and $R_{E/I}$ is the extravascular/intravascular ratio of binding proteins (usually 1.4 for albumin). V_P, V_E, and V_R refer to the volumes of plasma, extracellular fluid, and remainder fluid with values of 0.0436, 0.151, and 0.38 L/kg in humans; 0.0313, 0.265, and 0.364 L/kg in rats; 0.0515, 0.216, and 0.450 L/kg in dogs, respectively. This equation was rearranged to express f_{ut} in terms of V_{ss} and f_{up} as follows:

$$f_{ut} = \frac{V_R \times f_{up}}{[V_{ss} - V_p - (f_{up} \times V_E)] - [(1 - f_{up}) \times R_{E/I} \times V_p]}$$

The average f_{ut} of SD rats and beagle dogs were used as human f_{ut}.

3. Calculation of recommended doses of XZP-5610 in FIH clinical trial

Exposure-based method:

$$HED = AUC_{0\text{-}24h} \times \frac{f_{u,a}}{f_{u,h}} \times \frac{Cl_{i,v,h}}{F_a} \quad (11)$$

$$MRSD = HED \times SF \quad (12)$$

Where $AUC_{0\text{-}24h}$ represents animal steady-state AUC, $f_{u,a}$ represents animal plasma free fraction, $f_{u,h}$ represents human plasma free fraction, $CL_{i.v,h}$ represents human intravenous clearance rate, F_a represents animal bioavailability. *SF* is set as 10 in this study.

Body surface area-based method:

$$HED = NOAEL_a \times K \times BW_h \quad (13)$$

Where $NOAEL_a$ represents animal No Observed Adverse Effect Level, K represents coefficient, for rat is 0.16 and for monkey is 0.54, BW_h represents human body weight, which is 60 kg.

Table S1 Dose of XZP-5610 administrated to SPF SD rats in different groups in toxicity studies

Type of study	Administrated dose（mg/kg）			
	Group 1 (control)	Group 2 (low dose)	Group 3 (medium dose)	Group 4 (high dose)
Acute toxicity	0	500	1000	2000
long-term toxicity (Male)	0	1.5	4.5	15
long-term toxicity (Female)	0	1	3	10

Table S2 Dose of XZP-5610 administrated to beagle dogs in different groups in toxicity studies

Type of study	Administrated dose（mg/kg）			
	Group 1 (control)	Group 2 (low dose)	Group 3 (medium dose)	Group 4 (high dose)
Acute toxicity	0	125	250	500
long-term toxicity	0	0.05	0.2	0.8

Table S3 Half-life, intrinsic clearance and hepatic clearance of XZP-5610 in different species hepatocytes

Species	$T_{1/2}$ (min)	$CL_{int(hep)}$ (μL/min/10^6 cells)	$CL_{int(liver)}$ (mL/min/kg)	$CL_{(liver)}$ (mL/min/kg)
CD-1 mice	15.5	44.8	532.3	77.0
SD rat	17.8	38.8	181.7	42.3
Beagle dog	123.8	5.6	38.5	17.1
Cynomolgus monkey	78.2	8.9	31.9	18.5
Human	60.0	11.6	32.1	12.6

Table S4 Metabolites information of XZP-5610 in different species of hepatocytes

Name	Retention time (min)	Abundance (UV area%)				
		CD-1 mice	SD rat	Beagle dog	Cynomolgus monkey	Human
Parent	50.16	0.24	88.56	85.97	80.38	87.30
M1		ND				
M2	21.79	1.80	0.20	0.22	0.12	0.12
M3	33.09	ND	ND	ND	0.08	0.95
M4	43.57	0.22	3.39	0.57	0.85	1.82
M5	40.17	ND	3.83	12.74	17.91	9.78
M6						
M7	36.01	0.19	+	+	+	+
M8	38.46	0.68	2.58	ND	0.66	0.03
M9	29.29	4.26	ND	ND	ND	ND
M10	29.78	7.15	ND	+	+	ND

M11	30.61	0.79	ND	ND	+	ND
M12	32.61	65.77	1.43	0.50	ND	ND
M13	30.01	1.43	ND	ND	ND	ND
M14	35.23	4.48	ND	ND	ND	ND
M15	28.11	12.97	ND	ND	ND	ND
M16						

The data in the table represents semi-quantitative measurements of peak area at UV (315 nm);

+: Can be detected in MS and can't be detected in UV;

ND: not detected;

The peak of XZP-5610 and M1, M5 and M6, and M15 and M16 could not be separated in the LC-UV and the total abundance were presented in the table. The response of XZP-5610 in MS was 100 times higher than M1, so the main constitute in peak XZP-5610 and M1 should be the parent drug XZP-5610.

Table S5 Pharmacokinetic parameters after single administration of [^{14}C]XZP-5610 in rats

Tissue	T_{max} (h)	C_{max} (ng Eq/g)	AUC_{0-t} (h.ng Eq/g)
Fat	0.5	65.9	500
Heart	0.5	243	1249
Lung	0.5	304	724
Kidney	0.5	547	3430
Liver	0.5	13414	32765
Stomach	0.5	25175	56295
Intestine	2	6343	63911
Plasma	0.5	903	2722
Blood	0.5	486	972

Table S6 Body weight and biochemistry parameters changes in long-term toxicity studies of XZP-5610 in rats

Parameter [1]	Male (mg/kg) [2]			Female (mg/kg)		
	1.5	4.5	15	1	3	10
Body weight			4.7%↓			
ALB	12.7%↑	15.1%↑	22.5%↑	6.7%↑	7.6%↑	10.5%↑
A/G	7.0%↑	6.1%↑	7.8%↑			
ALP	49.0%↑	76.7%↑	149.2%↑			88.7%↑
TP	8.5%↑	11.1%↑	17.6%↑			10.1%↑
TCHO	40.3%↑		61.2%↑			34.0%↑
HDL	42.4%↑	65.4%↑	64.1%↑			37.6%↑
LDL	45.6%↑	44.9%↑	77.9%↑			

[1]: Serum biochemical indicators with statistical differences;

[2]: The changes percentage compared to the control group.

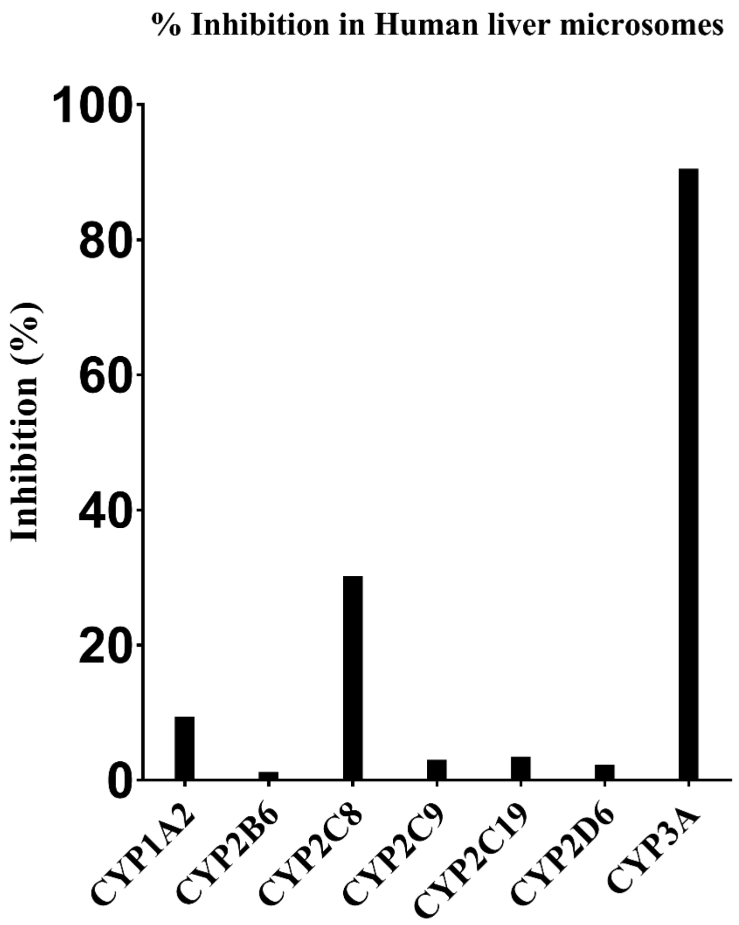

Figure S1 Inhibition of the metabolism of XZP-5610 in human liver microsomes by specific chemical inhibitors. Accordingly, the metabolism of XZP-5610 was not influenced by the inhibitors of CYP1A2, CYP2B6, CYP2C9, CYP2C19, and CYP2D6. Meanwhile, the metabolism of XZP-5610 could be slightly inhibited by the specific inhibitor of CYP2C8 and could be significantly inhibited by CYP3A inhibitor.
